# Response of Helicobacter Pylori Eradication Treatment in Patients With Normal and Below-Normal Serum Vitamin D Levels

**DOI:** 10.7759/cureus.14777

**Published:** 2021-04-30

**Authors:** Irshad Magsi, Shakir Hussain keerio, Chandar Kumar, Abdul Subhan Talpur, Fnu Shahzeen, Zohra Mushtaq Abbasi, Munisha Lohano, Vijay Kumar, Amber Rizwan

**Affiliations:** 1 Internal Medicine, Baqai Medical University, Karachi, PAK; 2 Internal Medicine, Liaquat University of Medical and Health Sciences, Jamshoro, PAK; 3 Medicine, Liaquat University of Medical and Health Sciences, Jamshoro, PAK; 4 Internal Medicine, Jinnah Sindh Medical University, Karachi, PAK; 5 Research and Statistics, Shaheed Zulfikar Ali Bhutto Institute of Science and Technology, Karachi, PAK; 6 Interventional Cardiology, Liaquat University of Medical & Health Sciences, Jamshoro, PAK; 7 Family Medicine, Jinnah Post Graduate Medical Center, Karachi, PAK

**Keywords:** vitamin d, treatment response, association, pakistan, helicobacter pylori

## Abstract

Introduction

The infection of *Helicobacter pylori* (*H. pylori*) is affected by the host immune system and the genetic makeup. It is postulated that deficiency of vitamin D may interfere in normal immunological response to infectious agents, including *H. pylori*, and increase the risk of infection. This study aims to find the relationship between vitamin D status in the body and patient’s response to *H. pylori* eradication treatment.

Methods

One hundred and fifty patients (n = 150) between the ages of 18 and 60 years of either gender, diagnosed with *H. pylori*, were included in the study. After enrollment, patients were started on first-line eradication therapy, which included omeprazole, amoxicillin, and clarithromycin for 14 days. Patient’s vitamin D levels were tested via laboratory. After 14 days, patients' stools were tested for presence of *H. pylori* antigen.

Results

A total of 128 participants completed the study, out of which 92 (71.8%) participants showed no *H. pylori* antigen in stool after 14 days and 36 (28.1%) participants still showed *H. pylori* in their stool. The mean serum vitamin D level was significantly higher in participants who had successful treatment compared to those who had unsuccessful treatment (31.01 ± 7.8 ng/mL vs. 18.9 ± 5.6 ng/mL; p-value < 0.0001).

Conclusion

Vitamin D levels may affect the response of *H. pylori* eradication therapy. Further large-scale studies are needed in which vitamin D is given as an intervention to further study the association between vitamin D levels and *H. pylori* treatment response.

## Introduction

*Helicobacter pylori* (*H. pylori*) is a spiral-shaped gram-negative microaerophilic bacterium first described in 1983 [1]. Today it affects almost 50% of the world’s population by colonizing the human gastric mucosa. It has been established as a major culprit of chronic gastritis and has also been identified to play an essential role in the pathogenesis of several other gastric conditions like peptic ulcer disease, gastric adenocarcinoma, and mucosa‐associated lymphoid lymphoma [2,3]. Almost 90% of duodenal ulcers and over three-fourths of gastric carcinomas are associated with *H. pylori* infections [4]. In patients with a positive family history of gastric cancers, the cancer incidence is seen to decline with the eradication of the infection [2,3].

Although *H. pylori* infection has a high global prevalence, it is inversely associated with socioeconomic development, more commonly affecting the developing world [5]. Like most other infectious diseases of the gastrointestinal tract, it also has an oral-fecal route of transmission [6]. The infection warrants timely treatment to hamper the recurrence and avoid the accompanying complications [7]. The preferred treatment, called triple therapy, includes one proton-pump inhibitor (PPI) with a combination of antibiotics like clarithromycin and amoxicillin (or metronidazole) [8]. The cure rate with triple therapy ranges from 75% to 85%; however, in recent times, this has fallen to a lower percent, attributed to the development of new antibiotic-resistant strains [9,10].

The infection of *H. pylori* is also affected by the host immune system and the genetic makeup [11]. Vitamin D, a widely recognized mediator of bone metabolism in the human body, also plays an important role in immunoregulation and inflammation. Studies have shown that vitamin D plays a vital part in the expression of genes coding for antimicrobial proteins (AMPs), which then modulate immune responses to various infections [12,13]. Therefore, the deficiency of vitamin D may be implicated to hamper the normal immunological response to infectious agents, including *H. pylori*, and increase the risk of infection [11,13]. As limited literature exists on the impact of vitamin D deficiency in *H. pylori* infections, this study aims to find the relationship between the vitamin D status in the body and patient’s response to *H. pylori* eradication treatment.

## Materials and methods

This study was conducted in the gastroenterology ward of a tertiary care hospital in Pakistan from March 2019 to January 2020. One hundred and fifty patients (n = 150) between the ages of 18 and 60 years of either gender diagnosed with *H. pylori* were included in the study. *Helicobacter pylori* was diagnosed with giemsa-stained gastric biopsy specimens. Patients already on antibiotics, PPI, or vitamin D supplements were not included in the study. Patients were enrolled via consecutive convenient non-probability sampling. After enrollment, patients were started on first-line eradication therapy, which included omeprazole, amoxicillin, and clarithromycin for 14 days. Patient's blood was drawn via phlebotomy and was sent to the laboratory for vitamin D levels. Vitamin D levels of less than 30 ng/mL were classified as hypovitaminosis [14]. Patients were followed up after 14 days and their stool was assessed for *H. pylori* antigen, using rapid test, to confirm eradication. If the antigen test was negative, treatment was labelled as successful. Compliance to medication was checked and confirmed with patients that if they have completed their treatment regime and no dose was missed. A total of 22 participants were lost to follow-up and only participants who completed the study were included in the final analysis.

Statistical analysis was done using Statistical Package for the Social Sciences (SPSS) v. 22.0 (IBM Corporation, Armonk, NY, United States). Continuous variables were tabulated as mean and standard deviation. Categorical variables were presented as percentages and frequencies. Independent t-test or chi square was applied as deemed appropriate. A p-value of less than 0.05 represented a difference between the two groups and the null hypothesis was void.

## Results

One hundred and twenty-eight (n = 128) participants completed the study. Ninety-two (71.8%) participants showed no *H. pylori* antigen in stool after 14 days, whereas 36 (28.1%) participants still showed *H. pylori* in their stool. Their demographics were comparable (Table 1). 

**Table 1:** Comparison of demographics of participants who underwent treatment

Demographics	Successful Treatment (n = 88)	Unsuccessful Treatment (n = 36)	p-Value
Gender			
Male	42 (47.7%)	18 (50.0%)	0.05
Female	46 (52.3%)	18 (50.0%)	
Age group (in years)			
18-30	21 (23.9%)	8 (22.2%)	0.94
31-40	32 (36.4%)	15 (41.6%)	
41-50	20 (22.7%)	8 (22.2%)	
51-60	15 (17.0%)	5 (13.8%)	

The mean serum vitamin D level was significantly higher in participants who had successful treatment compared to those who had unsuccessful treatment (31.01 ± 7.8 ng/mL vs. 18.9 ± 5.6 ng/mL; p-value < 0.0001). Number of participants with hypovitaminosis D were significantly more in the group with unsuccessful treatment (72.2% vs. 44.3%; p-value: 0.004) (Table 2).

**Table 2:** Outcome of treatment on the basis of vitamin D status of the patients ng, nanogram; mL, milliliter.

Vitamin D Status	Successful Treatment (n = 88)	Unsuccessful Treatment (n = 36)	p-Value
Mean vitamin D level (ng/mL)	31.01 ± 7.8	18.9 ± 5.6	<0.0001
No of participants with hypovitaminosis D	39 (44.3%)	26 (72.2%)	0.004

## Discussion

In our study, patients were treated to eradicate *H. pylori*. Women had a higher successful treatment rate (52.3%) as compared to men (47.7%). People belonging to the age group 31-40 were more likely to respond positively to the eradication therapy (36.4%), followed by the age group 18-30 (23.9%). Our study also demonstrated that successful treatment had a positive correlation with elevated vitamin D levels in the serum.

A number of studies provide evidence that vitamin D plays a major role in eradicating *H. pylori*. A study showed that vitamin D3 decomposition product (VDP1) selectively affects *H. pylori*, but did not affect the survival of *Enterobacteriaceae* bacteria, *Pseudomonas aeruginosa*, or *Staphylococcus aureus* in any way [15]. Several studies are of the idea that dimyristoyl phosphatidylethanolamine (DMPE), a common glycerophospholipid, is a major component of the cell membrane of *H. pylori* [16], and VDP1 results in dissolving bacteria by coming in contact with DMPE of *H. pylori* membrane. Another study proved that one of the products of vitamin D decomposition, alkyl of indene, interconnects with the di-14:0 DMPE of *H. pylori*. This leads to the breakdown, and the absence of alkyl proved the eradication of the germicidal effect on *H. pylori* [17]. Several studies suggested that serum vitamin D levels could potentially affect *H. pylori* eradication [18,19]. These studies provided evidence that decreased level of vitamin D is strongly associated with failure of *H. pylori* eradication.

The possible explanation of pathogenesis is that the close link between vitamin D level and eradication is that the disablement of vitamin D signals the immune system, causing insufficient immune response [8]. Apart from its effect on bones, vitamin D is known to decrease inflammatory markers like C-reactive protein, tumor necrosis factor-α, interleukin 6, and interleukin 18, and the level of anti-inflammatory cytokine interleukin 10 may increase [20]. It is also known to monitor the expression of AMPs cathelicidin and β-defensin, which are known to cause bacterial death. Therefore, it is said that low levels of vitamin D could result in failure of *H. pylori* due to reduced immune response, which make the conditions favorable for the bacteria. 

The study has its limitations as well. First, since it was a single center the sample was less diverse. Secondly, the impact of vitamin D supplement was not studied on eradication of *H. pylori*. Keeping in mind the aforementioned facts, further large-scale studies are required to explore vitamin D levels and its effect on *H. pylori* eradication. In cases of infection, vitamin D levels should be checked at regular intervals. In cases of its deficiency, it is advised to introduce vitamin D supplements to eliminate the chances of its deficiency worsening the cases. 

## Conclusions

Our study demonstrated that vitamin D levels in *H. pylori-*negative patients were higher than the *H. pylori-*positive patients. Furthermore, a positive correlation was found between successful eradication therapy and elevated vitamin D levels in the serum. Hence, we conclude that vitamin D levels may affect the response of *H. pylori* eradication therapy. Further large-scale studies are needed in which vitamin D is given as an intervention to further study the association between vitamin D levels and *H. pylori* treatment response, and to associate low vitamin D levels as a risk factor of treatment failure of *H. pylori*.
